# Fast, Simple, and Highly Specific Molecular Detection of *Porphyromonas gingivalis* Using Isothermal Amplification and Lateral Flow Strip Methods

**DOI:** 10.3389/fcimb.2022.895261

**Published:** 2022-05-25

**Authors:** Duobao Ge, Fang Wang, Yanyan Hu, Bendi Wang, Xuzhu Gao, Zhenxing Chen

**Affiliations:** Department of Stomatology, the Second People’s Hospital of Lianyungang City (Cancer Hospital of Lianyungang), affiliated to Bengbu Medical College, Lianyungang, China

**Keywords:** periodontal disease, *Porphyromonas gingivalis*, isothermal (DNA) amplification, lateral flow strip, rapid detection

## Abstract

*Porphyromonas gingivalis* is an important oral pathogen that causes periodontal disease and is difficult to culture under conventional conditions. Therefore, a reliable technique for detecting this pathogenic bacterium is required. Here, isothermal recombinase polymerase amplification (RPA), a new nucleic acid amplification method, was combined with a visualization method based on nanoparticle-based lateral flow strips (LFS) for the rapid detection of *P. gingivalis*. The species-specific 16S rRNA sequence of *P. gingivalis* was used as the target for RPA, and a set of specific primer–probe combinations were designed and screened to amplify the target sequences. As a thermostatic amplification method, the RPA reaction, under optimized conditions, takes only 30 min to complete at a constant temperature (37°C). The amplification reaction products can be detected visually by LFS without any need for special equipment. The RPA-LFS method established for the detection of *P. gingivalis* was shown to be highly specific in distinguishing *P. gingivalis* from other pathogenic organisms by using 20 clinical isolates of *P. gingivalis* and 23 common pathogenic microorganisms. Susceptibility measurements and probit regression analysis were performed with gradient dilutions of *P. gingivalis* genomic DNA. The method was obtained to be highly sensitive, with a detection limit of 9.27 CFU per reaction at 95% probability. By analyzing the gingival sulcus fluid specimens from 130 patients with chronic periodontitis, the results showed that the RPA-LFS method detected 118 positive cases and 12 negative cases of *P. gingivalis*, and the results obtained were consistent with those of a conventional PCR assay. The RPA–LFS method is an efficient, rapid, and convenient diagnostic method that simplifies the tedious process of detecting *P. gingivalis*.

## Introduction

Periodontal disease is a chronic infectious disease that occurs in the periodontal supporting tissues and is currently considered the sixth most prevalent disease in humans due to its high prevalence (Slots, 2017). Plaque microorganisms are known to be the initiating factors in the pathogenesis of periodontal disease, and among them, *Porphyromonas gingivalis* is currently recognized as the main dominant bacterium associated with the development of periodontal disease and is one of the key pathogenic bacteria studied in the field of periodontal microbiology (Olsen et al., 2017). *P. gingivalis* is a Gram-negative anaerobic bacterium that is present in the first complex (red complex) microflora of subgingival plaque in close association with chronic periodontitis (Zhang et al., 2020). As an opportunistic pathogen, *P. gingivalis* is not an aggressor of the inflammatory response, but rather a reciprocal reaction with the host that disrupts the host’s immune defense mechanisms, thereby prolonging its survival in the host and triggering the inflammatory response of the organism (Hajishengallis and Diaz, 2020). Previous studies have shown that *P. gingivalis* is closely associated not only with oral diseases such as aggressive periodontitis, periodontal abscesses and periapical inflammation (Mysak et al., 2014), but also with systemic systemic diseases such as atherosclerosis and gastrointestinal malignancies (Qi et al., 2020; Xie et al., 2020). In addition, *P. gingivalis* in periodontal pockets can be used to predict the progression of related diseases, and its number is significantly and positively correlated with the degree of disease (He et al., 2012; Yaseen et al., 2021). Therefore, exploring rapid detection methods for *P. gingivalis* are important guidelines for the diagnosis of oral diseases and related systemic diseases as well as for early intervention.

In the diagnosis of *P. gingivalis* infection, we currently rely on the traditional culture method for its detection, but this culture method requires the isolation, culture, and identification of the microorganism and has the disadvantages of being time-consuming, insensitive, and cumbersome (Atieh, 2008; Fyrestam et al., 2017). With the development of molecular biology techniques, rapid diagnostic studies of *P. gingivalis* have progressed rapidly, including the PCR detection of ribosomal RNA (16S rRNA), fluorescence quantitative PCR, immunochromatography, ionization time-of-flight mass spectrometry, and DNA probe hybridization, which can detect *P. gingivalis* rapidly at the gene or genome level (Rams et al., 2016; Mougeot et al., 2017; O'Brien-Simpson et al., 2017; Marin et al., 2018; Gu et al., 2020). However, these assays rely on trained technicians and sophisticated machines, which limits their application in remote areas and laboratories with limited resources. Therefore, a rapid, specific, sensitive, and device-independent detection method is urgently required (Qin et al., 2016; Alhogail et al., 2018; Ambrosio et al., 2019).

**Figure 1:**
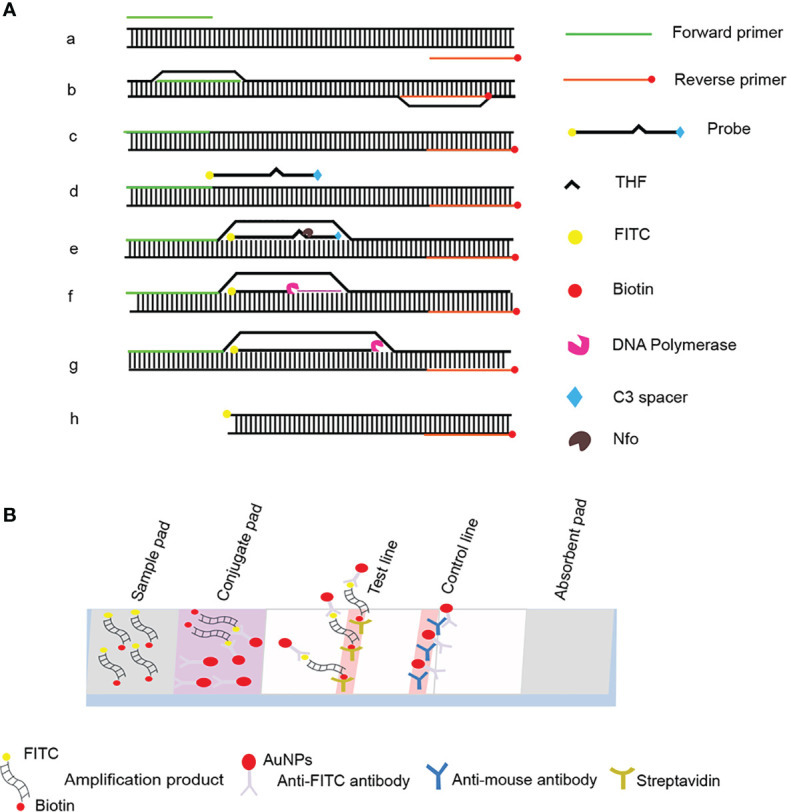
Schematic diagram of the RPA-LFS method. **(A)** Principle of RPA amplification. DNA strands are represented as horizontal lines, and base pairing is represented as short vertical lines between DNA strands. Base pairing is indicated as a short vertical line between DNA strands. **(B)** Schematic representation of the lateral flow strip (LFS) working principle. The shapes and their representative molecules are listed below the graphic.

The development of the recombinase polymerase amplification (RPA) technology, which is independent of complex laboratory instrumentation and expertise, has provided a molecular reference tool for the detection of *P. gingivalis* (James and Macdonald, 2015; Li et al., 2018). RPA mimics DNA replication in living organisms and achieves amplification at a constant temperature of 37°C to 42°C and is an alternative technique to PCR amplification (Jiang et al., 2020). In this case, recombinase binds to the primers to form stable nucleoprotein filaments that are used to find sites in the template sequence that are paired with the primer sequence. The double-stranded DNA is opened with the help of single-stranded binding proteins, and finally, the target region on the template is amplified exponentially by replicative extension with the action of Bsu DNA polymerase (Piepenburg et al., 2006; Lobato and O'Sullivan, 2018). The visual detection of labeled RPA amplification products by lateral flow strips (LFS) encapsulated with gold nanoparticles (AuNPs) further simplifies the detection process and allows on-site detection without instruments (**Figure 1**) (Li et al., 2019; Wang et al., 2021a).

In this study, a sensitive method for the rapid detection of *P. gingivalis* was developed using RPA combined with the LFS technology. The method was based on the 16S rRNA gene of *P. gingivalis*, which was used to design the primers and probes, and the assay could be completed for 30 min at 37°C. The specificity of the method was verified by testing 20 clinical isolates of *P. gingivalis* and 23 isolates of other common pathogens. The sensitivity of the RPA–LFS assay was determined in ten independent experiments, and the lowest limit of detection (LOD) was 9.27 colony-forming units (CFU)/reaction. Finally, the established *P. gingivalis* RPA–LFS assay was used to assay clinical specimens, and the results were accurate and consistent with those of the PCR method. In conclusion, in this study, we developed a rapid, sensitive, and specific RPA–LFS assay for *P. gingivalis*, which has promising applications for preliminary medical diagnoses in remote and resource-limited areas.

## Materials and Methods

### Ethical Statement

This study was approved by the Medical Ethics Committee of the Second People’s Hospital of Lianyungang City. One hundred and thirty patients with periodontitis in our hospital were enrolled (65 males and 65 females, aged 35–60 years). All subjects gave their written informed consent. Specimens of gingival sulcus fluid were collected with sterile absorbent paper tips inserted into periodontal pockets or gingival sulci and were sent to the laboratory for testing.

### Standard Strains and Clinical Isolates

A standard strain of *P. gingivalis* (ATCC 33277) was used to establish the RPA–LFS method for the detection of *P. gingivalis*. Twenty clinical isolates of *P. gingivalis* and 23 other common pathogens were collected. The latter included *Acinetobacter baumannii*, *Aggregatibacter actinomycetemcomitans*, *Bacillus cereus*, *Bacillus mirabilis, Burkholderia cepacia*, *Candida albicans*, *Candida tropicalis*, *Clostridium perfringens*, *Escherichia coli*, *Fusobacterium nucleatum*, *Haemophilus parainfluenzae*, *Klebsiella pneumoniae*, *Morganella morganii*, *Prevotella intermedia*, *Salmonella enterica*, *Serratia marcescens*, *Staphylococcus aureus*, *Stenotrophomonas maltophilia*, *Streptococcus mutans*, *Streptococcus pneumoniae*, *Streptococcus pyogenes*, *Tannerella forsythia*, and *Vibrio parahaemolyticus* were used to verify the specificity of the RPA–LFS method. All strains were provided by the Microbiology Department of the Second People’s Hospital of Lianyungang, and the identities of all were confirmed with rRNA sequencing (**Table 1**).

**Table 1:** Microbial isolates used in this study.

Species	Source	Strain amount
*Porphyromonas gingivalis*	ATCC 33277	1
*Acinetobacter baumannii*	Sputum	1
*Aggregatibacter actinomycetemcomitans*	Gingival sulcus fluid	1
*Bacillus cereus*	Urine	1
*Bacillus mirabilis*	Vaginal discharge	1
*Burkholderia cepacia*	Sputum	1
*Candida albicans*	Sputum	1
*Candida tropicalis*	Sputum	1
*Clostridium perfringens*	Blood	1
*Escherichia coli*	Sputum	1
*Fusobacterium nucleatum*	Gingival sulcus fluid	1
*Haemophilus parainfluenzae*	Sputum	1
*Klebsiella pneumoniae*	Sputum	1
*Morganella morganii*	Sputum	1
*Prevotella intermedia*	Gingival sulcus fluid	1
*Salmonella enterica*	Feces	1
*Serratia marcescens*	Sputum	1
*Staphylococcus aureus*	Sputum	1
*Stenotrophomonas maltophilia*	Sputum	1
*Streptococcus mutans*	Sputum	1
*Streptococcus pneumoniae*	Sputum	1
*Streptococcus pyogenes*	Sputum	1
*Tannerella forsythia*	Gingival sulcus fluid	1
*Vibrio parahaemolyticus*	Feces	1
*P. gingivalis*	Gingival sulcus fluid	20

### Extraction of Bacterial Genomes

For the reactions that used purified genomic DNA as the template, genomic DNA was extracted with the Bacterial Genomic DNA Extraction Kit (Tiangen Biochemical Technology Co., Ltd, Beijing, China) and stored at −20°C until further processing.

### Primer Design for RPA Reaction

Specific RPA primers were designed based on the *P. gingivalis* 16S rRNA gene sequence, using the Primer Premier 5 software. The primer design parameters were: primer size of 30–35 bp, product size of 100–500 bp, GC content of 20%–80%, and melting temperature (T_m_) of 50–100°C. All other parameters used were the default settings. Five primer pairs were selected for testing, based on the scores from high to low (General Biosystems Ltd., Anhui, China).

### RPA Procedure

To initially screen the forward and reverse primers, RPA amplification was performed with the TwistAmp Liquid DNA amplification kit (TwistDx Inc., Maidenhead, UK), according to the manufacturer’s instructions. Each 50 μL mixture contained 25 μL of 2 × Reaction buffer, 5 μL of 10 × Basic mix, 2.5 μL of 20 × Core mix, 2.1 μL of forward primers and 2.1 μL of reverse primer (both 10 μM), 1 μL of genomic DNA as template, and 9.8 μL of distilled water. To ensure that all the reaction systems reacted simultaneously, 2.5 μL of 280 mM magnesium acetate was added to the PCR tube cap, and was added to the reaction system simultaneously by transient centrifugation.

The reaction mixture was briefly centrifuged and then incubated in a heater at 37°C for 30 min. Reactions with distilled water as a template were used as negative controls. The amplification products were purified with a DNA purification kit (Tiangen, Beijing, China) and resolved with 1.5% agarose gel electrophoresis.

### RPA–LFS Probe Design

The Primer Premier 5 software was used to design specific probes that hybridized to sequences between the forward- and reverse-primer-targeting sequences, which should theoretically avoid, as far as possible, the formation of dimeric structures between the probe and the reverse primer (Daher et al., 2015). The criteria were: (1) probe size of 46–51 bp, GC content of 20%–80%, and T_m_ of 57–80°C; (2) maximum hairpin fraction set to 9, maximum primer–dimer fraction set to 9, maximum poly-X set to 5′, and all other parameters set to the default values; (3) 5′-end labeled with fluorescein isothiocyanate (FITC), and 3′-end blocked with a C3 spacer; and in the middle of the probe was replaced with a tetrahydrofuran (THF) group, with at least 30 bp before the THF site and at least 15 bp after it; and (4) the 5′-end of the reverse primer was labeled with biotin.

### RPA–LFS Procedure

To screen the probe and primer combinations, the RPA–LFS assay was performed with the TwistAmp DNA amplification kit (TwistDx Inc.), according to the manufacturer’s instructions. Each 50 μL reaction system contained 29.5 μL of hydration buffer, 2.1 μL each of the RPA forward and reverse primers (both 10 μM), 0.6 μL of RPA probe (10 μM), 11.2 μL of distilled water, 2 μL of genomic DNA, and dried enzyme pellets. To ensure that all the reaction systems started simultaneously, 2.5 µL of 280 mM magnesium acetate was added to the tube cap, transiently centrifuged, and immediately incubated at a constant temperature of 37°C for 30 min. An aliquot (5 µL) of the amplification product was used for visual inspection with LFS (Ustar BioTechnologies Ltd, Hangzhou, China) within 3 min. Two red lines may appear on the LFS, the control line was present in each test to ensure the validity of the LFS, whereas the test line was only observed in a positive reaction. Each sample had two bands, one for the sample itself and the other for the control.

### LOD Assay

A 10-fold series of dilutions of the *P. gingivalis* genome corresponding to 6 × 10^4^ CFU/μL to 6 × 10^−1^ CFU/μL, was prepared for the RPA–LFS assay. The minimum LOD of the method was determined with a probit regression analysis of 10 independent experiments.

### Evaluation of the Compliance Rate of RPA–LFS With PCR Method

Clinical specimens were collected from 130 patients with chronic periodontitis in the Department of Stomatology, Lianyungang Second People’s Hospital. Specimen collection: Patients were instructed to rinse their mouths with water, rinse with saline to remove food debris, and insert the tip of sterile absorbent paper into the periodontal pocket or gingival sulcus for 30 s. After 30 s, the paper was immediately removed and placed in an EP tube containing 1 mL of PBS (re-collected if blood was present). For DNA extraction, the specimens were centrifuged at 12,000 × g for 2 min, and the supernatant was discarded. 30 μL 5% Chelex-100 (Sigma, United States) was added to the precipitate, which was heat treated at 100°C for 10 min before serving as the templates. The PCR primers were designed according to the 16S rRNA sequence. The compliance rate of the RPA–LFS method used on the clinical specimens was evaluated by comparing the results with those of PCR. The compliance rate between two methods was calculated with the formula: ([number of positive samples detected with both methods + number of negative samples detected with both methods]/total number of samples) × 100%.

## Results

### Design and Screening of Primer Sets for RPA System

**Figure 2:**
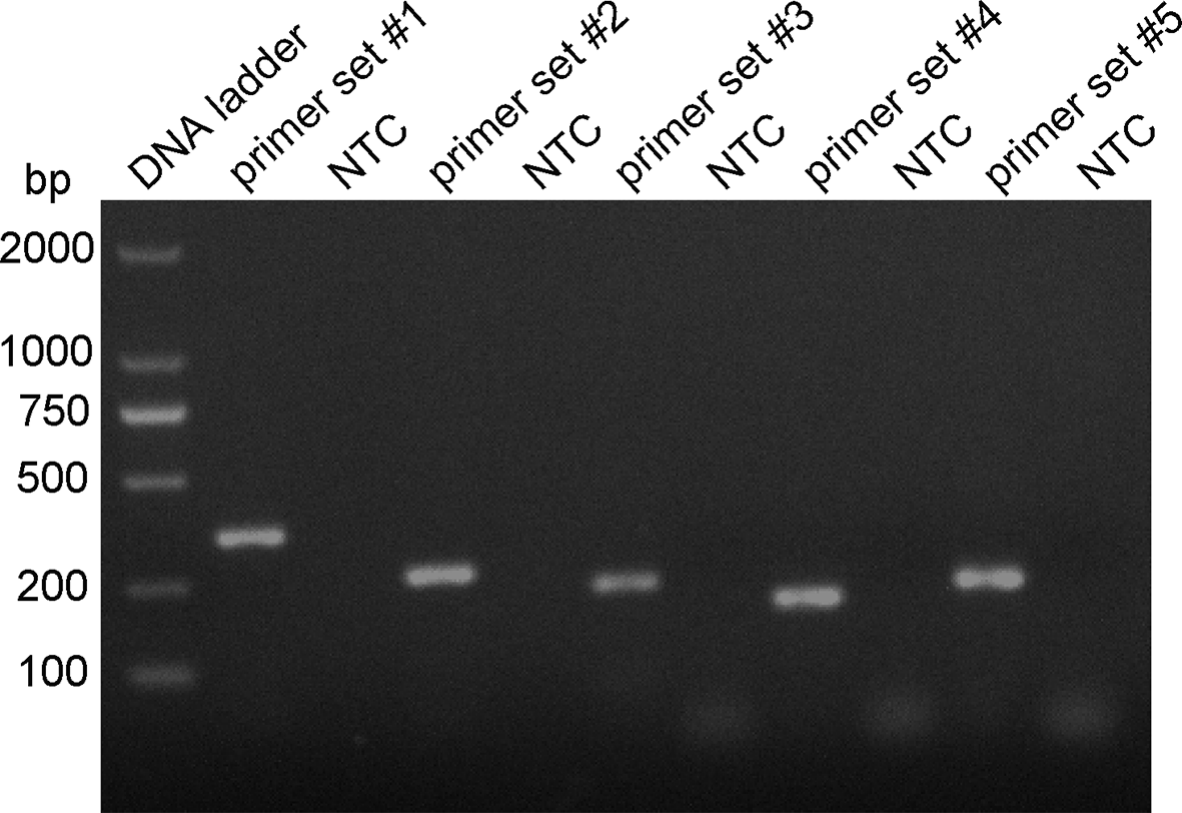
RPA primer set screening. The genomic DNA of the *P. gingivalis* standard strain was used as the template, and amplification with the RPA method was performed with primers set #1–5. The NTC strip is the no-template control for the corresponding RPA reaction, The amplification of the products was confirmed with 1.5% agarose gel electrophoresis.

Five primer pairs were designed using the 16S rRNA gene as the target sequence, and genomic DNA was used as the template for the RPA reaction (**Table 2**). All five primer pairs amplified the expected target bands with sizes of 353, 254, 223, 183, and 213 bp (**Figure 2**). Although there were no nonspecific amplified bands in the no-template controls (NTCs), primers 3, 4, and 5 showed primer dimers of < 100 bp. In contrast, primer set #1 and 2 amplified brighter target bands, with fewer primer dimers. Therefore, we selected primer set #1 and 2 for subsequent RPA–LFS probe design.

**Table 2:** Primers and probes tested in this study.

Name		Sequence (5’-3’)	Length (bp)	Amplicon size (bp)
# 1	Forward	AACGATGATTACTAGGAGTTTGCGATATAC	30	353
	Reverse	CCTTACGACGGCAGTCTCGGTAGAGTCCTCAGC	32	
# 2	Forward	ACCAAGGCGACGATGGGTAGGGGAACTGAG	30	251
	Reverse	GCTGCTGGCACGGAGTTAGCCGATGCTTATTC	32	
# 3	Forward	CCTGGTAGTCCACGCAGTAAACGATGATTA	30	220
	Reverse	TTCACCATCAGTCATCTACATTTCAATCCC	30	
# 4	Forward	CACCAAGGCGACGATGGGTAGGGGAACTGA	30	178
	Reverse	CCCGTATAAAAGAAGTTTACAATCCTTAGGAC	32	
# 5	Forward	CACCAAGGCGACGATGGGTAGGGGAACTGA	30	236
	Reverse	TAGCCGATGCTTATTCTTACGGTACATTCA	30	
P1	Forward	FITC-CCGTGAGGTGTCGGCTTAAGTGCCATAAC G[THF]GCGCAACCCACATCG-/C3-spacer/	46	95
P2	Forward	FITC-GGATTGTAAACTTCTTTTATACGGGAATA A[THF]GGGCGATACGAGTAT-/C3-spacer/	46	97
#1mR	Reverse	Biotin-TACCACGGCAGTCTCGGTAGAGTCCTCA GC	30	354
#2mR	Reverse	Biotin-GCTGCTGGCACGGAGTTAGCCGATGCTTCTTC	32	251
mP1	Forward	FITC-CCGCGATGTGTCGGATTAAGTGCCATAAC G[THF]GCGCAACCCACATCG-/C3-spacer/	46	95
mP2	Forward	FITC-GGATTGTAATCTTCTTTTATACGGGTATA A[THF]GGGCGATACGAGTAT-/C3-spacer/	46	97

Sequences modified with base substitutions. Modified bases are in red.

### Modification and Determination of Optimal Primer–Probe Combinations for RPA–LFS

**Figure 3:**
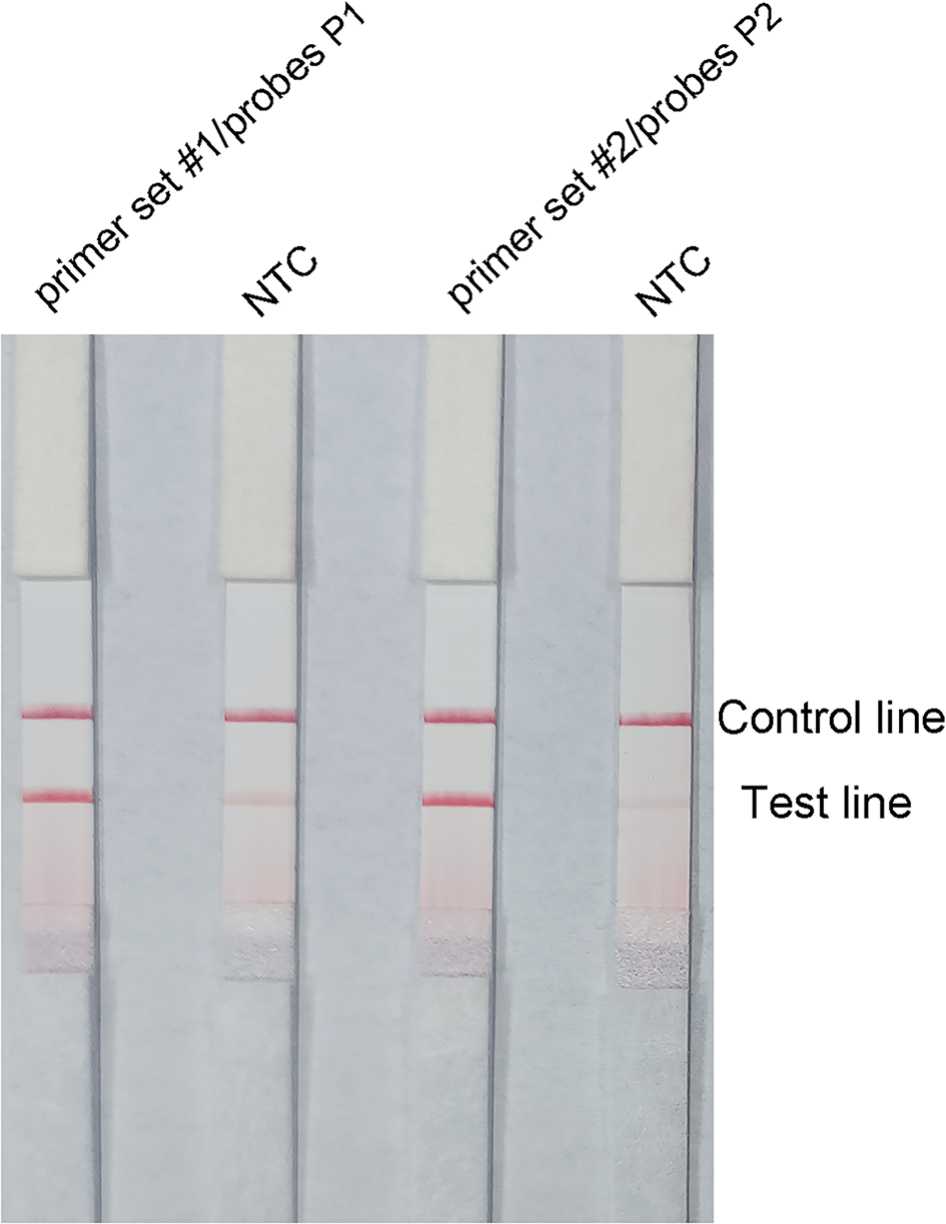
Performance of the primer–probe combinations tested with the RPA–LFS reaction. The results of LFS detection of the RPA amplification products are shown. The name of each primer–probe set is shown on the corresponding strip. The NTC strip is a template-free control for the individual RPA reaction. The reactions were performed for 30 min at 37°C. The positions of the test and control lines are marked on the right side of the bar graph. The image represents the results of three independent experiments.

Probes P1 and P2 were designed within the target sequences of primer set #1 and 2, respectively, and RPA–LFS assays were performed to test the amplification performance of the primer–probe combinations primer set #1/probes P1, primer set #2/probes P2, and the corresponding negative controls. Both primer–probe combinations generated the correct positive signals (two visible red bands on both the test and control lines), indicating that both primer–probe combinations performed well. However, the NTCs also showed a visible weaker red band on the test line, indicating a false-positive signal for both primer–probe combinations (**Figure 3**).

**Figure 4:**
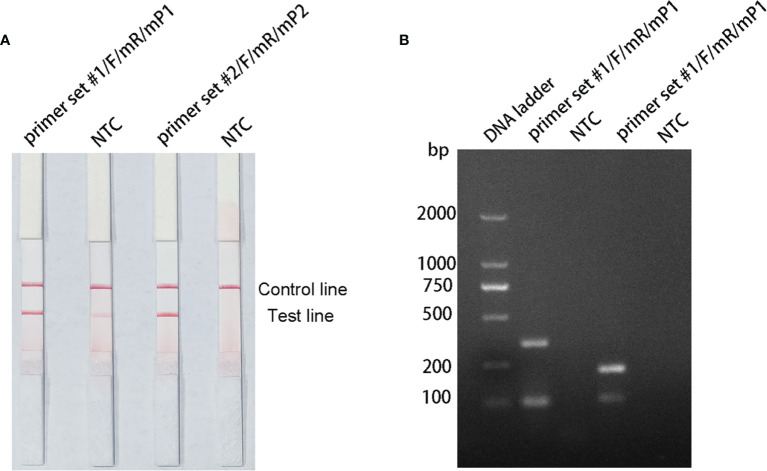
Testing the modified primer–probe combinations with the RPA–LFS reaction. **(A)** Graph shows the LFS results of RPA amplification. **(B)** The RPA amplification products were analyzed with agarose gel electrophoresis. The name of each primer–probe set is shown above the corresponding strip. The NTC strip is the no-template control for the corresponding RPA reaction, on the strip immediately to its left.

The pairing between of probe and the reverse primer may have been responsible for the false positive signals (Yang et al., 2021). The formation of a cross dimer between them was analyzed using the Primer Premier 5 software, and the results showed that both primer-probe pairs had multiple consecutive matching bases. Therefore, base substitution was introduced to eliminate consecutive base pairing between the probe and the reverse primer. The principles of this substitution were: (1) break was introduced into for sites with more than four consecutive matches or more than three consecutive matching bases at the 3′ end; and (2) the three bases near the 3′ end could not be replaced. The sequences of the modified reverse primers (mR) and probes (mP) are shown in **Table 2**, and the substituted bases are indicated in red. The RPA–LFS assay was performed using the modified primer and probe sets, but the results still showed a weak signal in the NTC set of the primer set #1/F/mR/mP1 combination. However, the primer set #2/F/mR/mP2 combination eliminated the weak false positive signal while not affecting amplification (**Figure 4A**). Analysis of the RPA amplification products with agarose gel electrophoresis revealed two clear bands for each of the two primer–probe combinations, representing the products of the forward and reverse primers and the probe and reverse primer (**Figure 4B**). Overall, the best primer–probe combination for the RPA–LFS assay of *P. gingivalis* was primer set #2/F/mR/mP2.

### Analysis of RPA–LFS Assay Specificity

**Figure 5:**
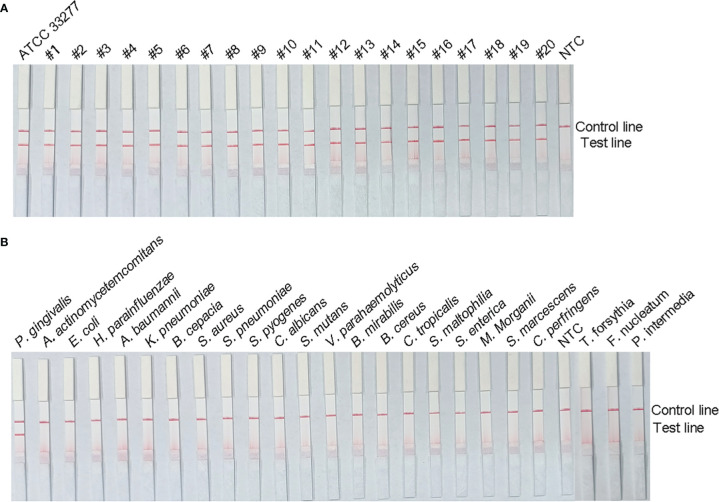
Specificity analysis of the *P. gingivalis* RPA–LFS assay. The specificity of the RPA–LFS assay established for *P. gingivalis* was tested on 20 clinical isolates of *P. gingivalis* **(A)** and genomic DNA extracted from 20 common pathogenic bacteria **(B)**. No-template control (NTC) was used as the negative control and *P. gingivalis* as the positive control. RPA amplification results were detected with LFS, and the samples are labeled at the top of the bar graph.

To verify the inclusiveness and specificity of the primer–probe combination, the RPA–LFS assay was used to analyze 20 clinical isolates of *P. gingivalis* and 23 isolates of other pathogenic bacteria. As shown in **Figure 5**, a clear positive signal appeared on the test line when isolated *P. gingivalis* genomic DNA was used as the template, but in contrast, no band appeared on the test line when genomic DNA from the other common pathogens was used as the template. These results demonstrate that the RPA–LFS assay system established is highly specific for *P. gingivalis*, with no cross-reactivity with other pathogens.

### Measurement of RPA–LFS Assay LOD

**Figure 6:**
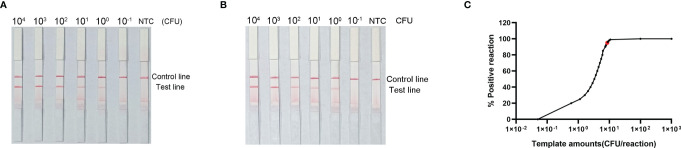
Determination of the limit of detection (LOD) of *P. gingivalis* RPA–LFS. **(A)** The LOD for the *P. gingivalis* RPA-LFS assay system established was determined from 10 independent assays using *P. gingivalis* genomic DNA at serial dilutions from 6 × 10^4^ to 6 × 10^−1^ CFU/μL. Images show the results of RPA–LFS, with the amount of template shown at the top of the strip. **(B)** The group with 10 ng human genomic DNA added in addition to the *P. gingivalis* genomic DNA. **(C)** Probit regression analysis of data collected from ten replicates, performed with the SPSS software.

To evaluate the LOD of the RPA–LFS assay, we used it to evaluate 10-fold dilutions of *P. gingivalis* genomic DNA, ranging from 6 × 10^4^ CFU/μL to 6 × 10^−1^ CFU/μL (1 μL in a 50 μL reaction volume) as the template. There was a clear red band on the test line at 10^4^ CFU/μL, and the signal diminished as the template concentration decreased. The signal disappeared in the 6 × 10^−1^ CFU/μL group (**Figure 6A**). To test whether the system was resistant to interference from human genomes, 10 ng of human DNA were added to the RPA reaction along with dilutions of *P. gingivalis* genomic DNA. The detection sensitivity was not affected by human DNA (**Figure 6B**). In addition, not all assays yielded positive results when strains with concentrations of 6 × 10^0^ CFU/μL (seven positive results from ten samples, 7/10) or 6 × 10^−1^ CFU/μL (1/10) were used as templates. To more accurately determine the LOD of the RPA–LFS assay, a probit regression analysis was performed on data from ten independent assays; the statistical analysis was performed with SPSS software. With 95% probability, the LOD for the reaction was 9.27 CFU/μL (**Figure 6C**).

### Application of *P. gingivalis* RPA–LFS to the Analysis of Clinical Specimens

To evaluate the clinical utility of the established RPA–LFS detection system, gingival sulcus fluid specimens from 130 patients with chronic periodontitis were tested with both the RPA–LFS method and PCR. As shown in **Table 3**, 118 samples positive for *P. gingivalis* and 12 negative samples were detected with the RPA–LFS method, and the results obtained were in complete agreement with those of the PCR method. These results suggest that the highly specific and sensitive *P. gingivalis* RPA-LFS is feasible and reliable when applied to clinical samples from patients.

**Table 3:** Analysis of gingival sulcus fluid specimens from 130 patients with chronic periodontitis using the RPA–LFS method and PCR.

Method	positive	negative	Amplification time (min)
RPA-LFS	118	12	35
qPCR	118	12	90

## Discussion

The isothermal amplification method RPA can rapidly amplify target DNA under low-temperature isothermal conditions with a tolerance for unpurified samples, and is therefore a promising alternative molecular assay for the detection of *P. gingivalis*. The chemical labeling of the RPA reaction allows the products to be read with an AuNP-based LFS, with less reliance on equipment and trained personnel. However, all these simplifications mean that the inherent risk of false-positive signals from primer dimers is not negligible (Miao et al., 2019). It has been reported that the introduction of probes into RPA reactions can reduce primer-dependent artifacts to some extent, but the introduction of probes alone does not guarantee the complete elimination of false-positive signals (Wu et al., 2020). The probe sequence can still pair with the reverse primer to some extent. We took advantage of the fact that the RPA reaction can tolerate some primer-to-template base mismatches and substituted some bases in the probe and reverse primer of RPA–LFS (Wang et al., 2021b). An improved RPA–LFS system was established by rigorously testing the efficacy of these measures, which completely prevented the formation of probe–primer complexes and eliminated false-positive signals.

Increased sensitivity is recognized as a key factor in the development of diagnostic methods. To determine the accurate LOD of the *P. gingivalis* RPA–LFS, we assayed different amounts of *P. gingivalis* genomic DNA template, ranging from 6 × 10^4^ CFU to 6 × 10^−1^ CFU. According to a probit regression analysis, the LOD of the *P. gingivalis* RPA–LFS was 9.27 CFU per reaction, with 95% probability, which is similar to the LOD of other highly sensitive molecular assays, including multiplex qPCR (50 pg), single PCR with purified DNA (0.5 pg) or crude bacterial cultures (10 CFU), and loop-mediated isothermal amplification (1.4 × 10^-1^ pg/μL) (Coffey et al., 2016; Su et al., 2019; Rao et al., 2021).

Early chairside microbiological tests used to screen for periodontitis risk included the use of Nbenzoyl-DL-arginine-2-naphthylamide (BANA) tests to determine the level of protease activity in subgingival plaque, agglutination of latex beads and whole-cell bacterial ELISA (Nisengard et al., 1992; Boyer et al., 1996; Loesche et al., 1997). In most of these previous tests, high sensitivity was achieved at the expense of specificity (Hemmings et al., 1997; Eick and Pfister, 2002; Kaman et al., 2012). The immunochromatographic device using A1-adhesin monoclonal antibodies has emerged in recent years, and the sensitivity and specificity of the current assay device are 95.0% and 93.3%, respectively, which is a great improvement compared to the previous ones. However, this method requires ELISA and microbial flow cytometry to screen for specific monoclonal antibodies, and the whole operation is complicated and costly (O'Brien-Simpson et al., 2017). *In vitro* isothermal nucleic acid amplification strategies are important in molecular diagnosis, and loop-mediated isothermal amplification (LAMP) is one of the more widely used ones. Su et al. used LAMP method to detect *P. gingivalis* against specific fragments with a lower limit of detection of 1.4 × 10^-1^ pg/μl, and the method was non-cross-reactive with other bacterial pathogens (Su et al., 2019). However, LAMP typically has a reaction temperature of 60-65°C and requires three primer pairs, which may lead to primer-primer interactions, thus limiting the reaction.

LFS-based tests are considered the gold standard for point-of-care diagnostics and are portable, rapid, and simple to use. This study was based on the visualization of RPA with the LFS technology, using the 16S rRNA sequence as the target, and the assay was completed within 30 min under isothermal conditions at 37°C. A comparison of clinical isolates of *P. gingivalis* with those of other common pathogenic bacteria demonstrated the excellent specificity of the method. When the RPA–LFS assay was used to test clinical specimens, the samples did not require purification and could be used for the assay after simple processing. The assay showed 100% accuracy, in agreement with the conventional PCR method. In conclusion, in this study, we have established a rapid, specific, and sensitive field assay for *P. gingivalis*. Readable results can be obtained within 1 h with a simple procedure under equipment-free conditions. This may provide a reference for future chairside rapid detection of *P. gingivalis* and other pathogenic microorganisms, and will also contribute to the early diagnosis of related diseases, which is an important guide for early intervention and clinical treatment of oral diseases and systemic diseases.

## Data Availability Statement

The original contributions presented in the study are included in the article/supplementary material. Further inquiries can be directed to the corresponding authors.

## Ethics Statement

This study was approved by the Medical Ethics Committee of the Second People’s Hospital of Lianyungang City. One hundred and thirty patients with periodontitis in our hospital were enrolled (65 males and 65 females, aged 35–60 years). All subjects gave their written informed consent. Specimens of gingival sulcus fluid were collected with sterile absorbent paper tips inserted into periodontal pockets or gingival sulci and were sent to the laboratory for testing.

## Author Contributions

DG and FW conceived and designed all the experiments and wrote the paper. XG suggested the primer–probe design and directed the experiments. YH collected the clinical specimens, and BW analyzed the data. ZC revised the manuscript, submitted the manuscript, and led all the work. All the authors discussed the results, provided comments, and approved the final version of the manuscript.

## Funding

This work was funded by the High-level Innovation and Entrepreneurship Talents Introduction Program of Jiangsu Province of China (grant number 2019-30345), the ‘521 Project’ scientific research funding project of Lianyungang City (grant number LYG06521202157), the ‘HaiYan Plan’ scientific research funding project of Lianyungang City (grant number 2019-QD-008), and the Clinical Medical Science and Technology Development Fund of Jiangsu University (grant number JLY20180020).

## Conflict of Interest

The authors declare that the research was conducted in the absence of any commercial or financial relationships that could be construed as a potential conflict of interest.

## Publisher’s Note

All claims expressed in this article are solely those of the authors and do not necessarily represent those of their affiliated organizations, or those of the publisher, the editors and the reviewers. Any product that may be evaluated in this article, or claim that may be made by its manufacturer, is not guaranteed or endorsed by the publisher.
